# Development of SARS-CoV2 humoral response including neutralizing antibodies is not sufficient to protect patients against fatal infection

**DOI:** 10.1038/s41598-022-06038-5

**Published:** 2022-02-08

**Authors:** Mathilde Choteau, Anaïs Scohy, Stéphane Messe, Mathieu Luyckx, Mélanie Dechamps, Virginie Montiel, Jean Cyr Yombi, Damien Gruson, Nisha Limaye, Thomas Michiels, Laure Dumoutier

**Affiliations:** 1 Experimental Medicine Unit, de Duve Institute, Université Catholique de Louvain, 74 Avenue Hippocrate, 1200 Brussels, Belgium; 2 Department of Laboratory Medicine, Cliniques Universitaires Saint-Luc, Brussels, Belgium; 3 Institut de Recherche Expérimentale et Clinique (IREC), Université Catholique de Louvain, Brussels, Belgium; 4 Virology Unit, de Duve Institute, Université Catholique de Louvain, Brussels, Belgium; 5 Department of Gynecology-Andrology, Cliniques Universitaires Saint-Luc, Université Catholique de Louvain, Brussels, Belgium; 6 Tumor Infiltrating Lymphocytes Unit, de Duve Institute, Université Catholique de Louvain, Brussels, Belgium; 7 Pôle de Recherche Cardiovasculaire (CARD), Institut de Recherche Expérimentale et Clinique (IREC), Université Catholique de Louvain (UCLouvain), Brussels, Belgium; 8 Cardiovascular Intensive Care, Cliniques Universitaires Saint-Luc, Brussels, Belgium; 9 Intensive Care, Cliniques Universitaires Saint-Luc, Brussels, Belgium; 10 Pôle de Recherche Pharmacologie et de Thérapeutique (FATH), Institut de Recherche Expérimentale et Clinique (IREC), Université Catholique de Louvain (UCLouvain), Brussels, Belgium; 11 Department of Internal Medicine and Infectious Diseases, Cliniques Universitaires Saint-Luc, Brussels, Belgium; 12 Genetics of Autoimmune Diseases and Cancer, de Duve Institute, Université Catholique de Louvain, Brussels, Belgium

**Keywords:** Immunological disorders, Antibodies

## Abstract

More than a year after the start of the pandemic, COVID-19 remains a global health emergency. Although the immune response against SARS-CoV-2 has been extensively studied, some points remain controversial. One is the role of antibodies in viral clearance and modulation of disease severity. While passive transfer of neutralizing antibodies protects against SARS-CoV-2 infection in animal models, titers of anti-SARS-CoV-2 antibodies have been reported to be higher in patients suffering from more severe forms of the disease. A second key question for pandemic management and vaccine design is the persistence of the humoral response. Here, we characterized the antibody response in 187 COVID-19 patients, ranging from asymptomatic individuals to patients who died from COVID-19, and including patients who recovered. We developed in-house ELISAs to measure titers of IgG, IgM and IgA directed against the RBD or N regions in patient serum or plasma, and a spike-pseudotyped neutralization assay to analyse seroneutralization. Higher titers of virus-specific antibodies were detected in patients with severe COVID-19, including deceased patients, compared to asymptomatic patients. This demonstrates that fatal infection is not associated with defective humoral response. Finally, most of recovered patients still had anti-SARS-CoV-2 IgG more than 3 months after infection.

## Introduction

Although differences in susceptibility to SARS-CoV-2 may be highly complex and multifactorial, age, comorbidities, expression levels of SARS-CoV-2 receptors and host immune responses are likely strong determinants of outcome^1,2^. Most COVID-19 patients are reported to produce antibodies directed against the spike (S) and nucleocapsid (N) proteins, which seem to be the main viral immunogens^3–5^. While the N protein holds the viral RNA genome, the S protein is exposed at the surface and is responsible for viral entry via direct contact between its receptor binding domain (RBD) and the human receptor angiotensin-converting enzyme 2 (ACE2)^6,7^. The S protein is therefore more prone to seroneutralization and numerous neutralizing anti-RBD monoclonal antibodies have been characterized^8–13^. Nevertheless, Albecka et al. showed that anti-N antibodies can neutralize SARS-CoV-2 intracellularly and this mechanism requires the cytosolic Fc receptor and E3 ubiquitin ligase, TRIM21^14^. Overall, the seroconversion times for IgA, IgM, and IgG are about 4–6, 4–6, and 5–10 days post-symptom onset (PSO). Neutralizing antibodies were shown to be associated with protective immunity against secondary infection with SARS-CoV-2 in animal models^15–17^. However, the role of antibodies in modulation of disease severity in patients remains controversial.

Some studies have shown that titers of anti-SARS-CoV-2 antibodies appear earlier and are more elevated in patients suffering from severe forms of the disease compared with mild or asymptomatic cases, raising the possibility of a pathological role of antibody response^18,19^. In contrast, other reports have shown a correlation between antibody titers and a positive outcome^20^. It was reported that deceased patients did not have higher anti-spike IgG, anti-RBD IgG, and neutralizing antibodies, and mounted a robust but delayed response compared to survivors. In addition, the generation of neutralizing antibodies within 14 days of disease onset seems to be a key factor for recovery^20^. A recent study also measured SARS-CoV-2-specific IgA antibodies in the serum, saliva, and bronchoalveolar fluid of patients with COVID-19 and showed that IgA antibodies are predominant in the early phase of SARS-CoV-2 infection^21^. Moreover, IgA from serum and mucosal surfaces contributes to virus neutralization to a greater extent than IgG. Together, these studies argued against a simple correlation of SARS-CoV-2-specific antibody titers with disease severity. Instead, seroconversion kinetics, antibody isotype, and antigen specificity of antibodies should also be considered in determining the effect of the humoral response on disease severity.

The longevity of the immune memory response is critical for protection from pathogen reinfection. There are several controversial reports about the persistence of humoral responses against SARS-CoV-2^22–29^. Some studies have shown stable antibody levels for several months^27–29^, whereas others have described a rapid decline of anti-SARS-CoV-2 IgG by 3 months^25,26^. Interestingly, the waning of anti-SARS-CoV-2 IgA antibodies seems to be less rapid than that of other isotypes including IgM and IgG^30^. In some studies, a rapid decline in antibody titers against SARS-CoV-2 was found in COVID-19 patients with mild or no symptoms, suggesting that the longevity of the antibody response may correlate with disease severity^23,24^.

A good understanding of humoral responses to SARS-CoV-2 is essential for the management of the pandemic and the monitoring of vaccines. In this study, we characterized the humoral response in 187 COVID-19 patients classified by severity of symptoms and outcome: asymptomatic individuals, hospitalized individuals who were admitted to intensive care unit (ICU) or not and who survived, hospitalized individuals who died due to the infection, and individuals who recovered from the infection without having undergone hospitalization. We developed a spike-pseudotype neutralization assay and in-house enzyme-linked immunosorbent assays (ELISAs) to measure seroneutralization and titers of IgG, IgM and IgA directed against RBD or N protein. We detected higher titers of virus-specific antibodies in hospitalized (non-ICU or ICU survivors and deceased) patients compared to asymptomatic patients. In addition, seroneutralization capacity correlated with disease severity. More than 60 days PSO, few patients had anti-SARS-CoV-2 IgM and IgA, while the majority still had anti-SARS-CoV-2 IgG.

## Results

### Study cohorts

We evaluated the humoral response to SARS-CoV-2 in five different cohorts of COVID-19 patients: (1) asymptomatic COVID-19 patients (n = 34), (2) hospitalized non-ICU COVID-19 patients (n = 44), (3) ICU COVID-19 patients (n = 33), (4) deceased COVID-19 patients (n = 23) and (5) non-hospitalized, recovered COVID-19 patients (n = 53). All patients tested positive for SARS-CoV-2 by RT-PCR on nasopharyngeal swab specimens except asymptomatic patients, who were selected based on serological screening for anti-SARS-CoV-2 antibodies. Non-ICU and ICU COVID-19 patients were hospitalized due to lung failure in a conventional unit or the intensive care unit, respectively, of Cliniques universitaires Saint-Luc. All these patients survived their disease. To study the association between antibody response and mortality, we included ICU patients who died due to COVID-19. Serum or plasma of hospitalized non-ICU, ICU and deceased COVID-19 patients were collected between 2 and 27 days PSO. In order to analyse the longevity of anti-SARS-CoV-2 antibodies, we also included non-hospitalized patients who had mild to moderate COVID-19 more than 61 days earlier and had recovered. The samples were collected between March and November 2020, so that the original strain from Wuhan almost certainly accounts for the vast majority if not all. Samples from healthy individuals (17 pre-pandemic and 35 post-pandemic) were used as negative controls. Clinical characteristics of each cohort are shown in Table 1. Hospitalized patients (non-ICU, ICU or deceased) were mostly men with a mean age of over 59 years, which increased with patient disease severity. Conversely, healthy individuals, asymptomatic and non-hospitalized recovered COVID-19 patients were mostly female with a mean age of 37–40 years depending on cohort.

**Table 1:** Clinical characteristics of the six cohorts.

Characteristic	Healthy individuals (n = 52)		Asymptomatic COVID-19 patients (n = 34)		Non-ICU COVID-19 patients (n = 44)		ICU COVID-19 patients (n = 33)		Deceased COVID-19 patients (n = 23)		Recovered COVID-19 patients (n = 53)	
	n	%	n	%	n	%	n	%	n	%	n	%
**Age (in years), mean**	37*		38		59		64		70		40	
**Sex**												
Male	17	32.69%	7	20.59%	24	54.55%	26	78.79%	17	73.91%	11	20.75%
Female	15	28.85%	27	79.41%	16	36.36%	4	12.12%	6	26.09%	40	75.47%
Unkown	20	38.46%	0	0.00%	4	9.09%	3	9.09%	0	0.00%	2	3.77%
**Days post symptom onset**												
0–4	N/A	N/A	N/A	N/A	6	13.64%	1	3.03%	2	8.70%	0	0.00%
5–9	N/A	N/A	N/A	N/A	13	29.55%	10	30.30%	8	34.78%	0	0.00%
10–15	N/A	N/A	N/A	N/A	21	47.73%	17	51.52%	4	17.39%	0	0.00%
15–27	N/A	N/A	N/A	N/A	4	9.09%	5	15.15%	7	30.43%	0	0.00%
61–75	N/A	N/A	N/A	N/A	0	0.00%	0	0.00%	0	0.00%	5	9.43%
76–90	N/A	N/A	N/A	N/A	0	0.00%	0	0.00%	0	0.00%	20	37.74%
91–105	N/A	N/A	N/A	N/A	0	0.00%	0	0.00%	0	0.00%	23	43.40%
Unknown	N/A	N/A	N/A	N/A	0	0.00%	0	0.00%	2	8.70%	5	9.43%
**Reported symptoms**												
Fever	0	0.00%	0	0.00%	29	65.91%	20	60.61%	12	52.17%	34	64.15%
Cough	0	0.00%	0	0.00%	22	50.00%	18	54.55%	10	43.48%	35	66.04%
Dyspnea	0	0.00%	0	0.00%	26	59.09%	16	48.48%	18	78.26%	32	60.38%
Anosmia	0	0.00%	0	0.00%	6	13.64%	2	6.06%	2	8.70%	37	69.81%
Agueusia	0	0.00%	0	0.00%	9	20.45%	3	9.09%	1	4.35%	37	69.81%
Tiredness	0	0.00%	0	0.00%	27	61.36%	19	57.58%	11	47.83%	NA	NA
Rhinitis	0	0.00%	0	0.00%	6	13.64%	2	6.06%	2	8.70%	NA	NA
Headache	0	0.00%	0	0.00%	8	18.18%	5	15.15%	0	0.00%	NA	NA
Myalgia	0	0.00%	0	0.00%	10	22.73%	12	36.36%	4	17.39%	37	69.81%
Diarrhea	0	0.00%	0	0.00%	4	9.09%	2	6.06%	1	4.35%	26	49.06%
Nausea/vomiting	0	0.00%	0	0.00%	3	6.82%	1	3.03%	1	4.35%	NA	NA

*Data available for 32 cases.

*NA* not available.

*N/A* not applicable.

### High humoral response in patients with severe forms of COVID-19

**Figure 1:**
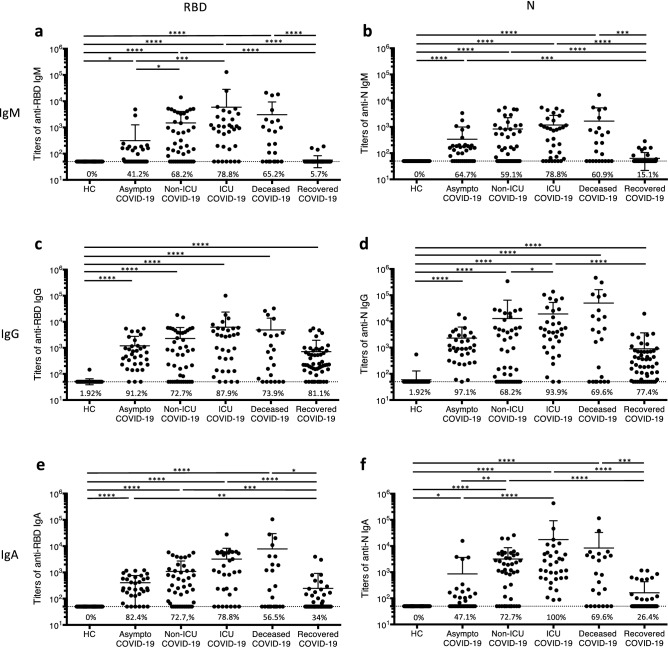
Antibody responses against SARS-CoV-2 in COVID-19 patients. Titers of IgM (**a**,**b**), IgG (**c**,**d**) and IgA (**e**,**f**) directed against RBD (left panels) or N (right panels) in serum or plasma of healthy controls and COVID-19 patients were determined by ELISA. The six patient cohorts are presented in Table 1. The percentage of positive samples is indicated. Samples were considered positive when antibody titer was greater than 50 (dashed line), the lowest dilution of serum/plasma tested. Each dot represents one sample. *p < 0.05, **p < 0.01, ***p < 0.001, ****p < 0.0001 (Kruskal Wallis test and Dunn’s multiple comparisons test).

To characterize the humoral immune response across these cohorts, we generated an ELISA to detect IgM, IgG and IgA directed against N or RBD. Specificity of the ELISA was demonstrated by failure to detect anti-SARS-CoV-2 IgM and IgA in healthy controls, and the low percentage of this group in whom anti-SARS-CoV-2 IgG was detected (1.92%, 1 out of 52 healthy controls, Fig. 1a–f). Anti-RBD and anti-N IgM were detected in 41.2% and 64.7% respectively of samples from asymptomatic patients. The percentage of patients with detectable anti-SARS-CoV-2 IgM was 78.8% in ICU COVID-19 cohort, and titers were also higher than in other groups. In agreement with their short lifespan, anti-SARS-CoV-2 IgM were detected in few samples from recovered patients (15.1% for anti-N IgM and 5.7% for anti-RBD IgM) and titers of these antibodies were close to the detection threshold (Fig. 1a,b). Almost all asymptomatic patients had anti-RBD IgG (91.2%) and anti-N IgG (97.1%), as expected based on the recruitment criteria. However, titers were lower in asymptomatic than in hospitalized patients, in whom anti-N IgG levels increased with disease severity. The majority of recovered patients still had anti-SARS-CoV-2 IgG (81.1% with anti-RBD IgG and 77.4% with anti-N IgG) more than 2 months after infection, highlighting the persistence of these antibodies (Fig. 1c,d). The titers of anti-SARS-CoV-2 IgA, which would play an important role in the defense against mucosal infection, showed a similar distribution between groups as the other two isotypes. The highest titers of anti-SARS-CoV-2 IgA were observed in ICU COVID-19 patients and deceased patients. Of note, 100% of ICU COVID-19 patients had anti-N IgA, demonstrating that these antibodies do not prevent the development of a severe form of the disease. Conversely, anti-RBD IgA were detected in twice as many asymptomatic patient samples as anti-N IgA. More than 2 months after infection, anti-SARS-CoV-2 IgA persists in about one third of recovered patients (Fig. 1e,f).

### Positive correlation between neutralization capacity and disease severity

**Figure 2:**
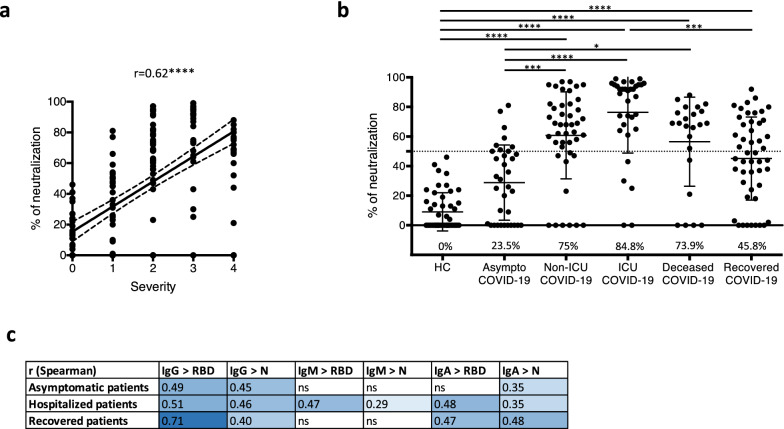
Correlation of spike-pseudotyped lentiviral neutralization, disease severity and serology results. (**a**) COVID-19 patients were classified according to their disease severity (0 = healthy controls, 1 = asymptomatic COVID-19 patients, 2 = hospitalized non-ICU COVID-19 patients, 3 = ICU COVID-19 patients, 4 = deceased COVID-19 patients). Correlation between spike-pseudotyped lentiviral neutralization and severity was assessed with simple linear regression (solid line) and 95% confidence bands (dashed line) of the best-fit line. The Spearman’s correlation coefficient (r) is shown. (**b**) The neutralization capacity in serum or plasma of healthy controls and COVID-19 patients was determined by spike-pseudotyped lentiviral neutralization assay and was expressed in terms of a percentage of neutralization. The six patient cohorts are presented in Table 1. The percentage of neutralizing samples is indicated. Samples were considered neutralizing when neutralization capacity was greater than 50% (dashed line). (**a**,**b**) Each dot represents one sample. *p < 0.05, ***p < 0.001, ****p < 0.0001 (Kruskal Wallis test and Dunn’s multiple comparisons test). (**c**) Correlations between spike-pseudotyped lentiviral neutralization and titers of IgG, IgM and IgA directed against RBD or N were assessed for asymptomatic COVID-19 patients, hospitalized COVID-19 patients (including non-ICU, ICU and deceased COVID-19 patients) and recovered patients. Spearman’s correlation coefficients (r) are shown and visualized by colour intensity. All correlations are significant (*p* < 0.05) except those indicated by "ns".

Overall, levels of anti-SARS-CoV-2 antibodies were higher in patients with more severe COVID-19 (hospitalized patients including non-ICU, ICU and deceased patients), bringing into question the efficiency of the humoral response in controlling the infection. We developed a lentiviral SARS-CoV-2 spike-pseudotype neutralization assay to test for the neutralizing capacity of patient serum or plasma. This may provide a more pertinent estimation of in vivo humoral immunity than simply titrating antibodies. Strikingly, we showed that neutralization capacity correlated with the severity of patient disease with a Spearman coefficient of 0.62 (Fig. 2a). The percentage of serum or plasma samples with > 50% neutralization capacity was high in ICU COVID-19 patients and in deceased patients (84.8% and 73.9%, respectively) whereas the percentage of neutralizing samples was only 23.5% in the asymptomatic group, despite its high seropositivity for anti-SARS-CoV-2 IgG. Half of the patients who recovered from mild to moderate COVID-19 without hospitalization had neutralizing antibodies more than 2 months after infection (Fig. 2b). In these patients, the neutralization capacity correlated with anti-RBD IgG with a Spearman coefficient of 0.71 (Fig. 2c). Taken together, these results demonstrate that humoral response, even with neutralizing antibodies, is not sufficient to prevent the development of a severe form of COVID-19.

### Kinetics of anti-SARS-CoV-2 antibody production is similar in deceased patients

**Figure 3:**
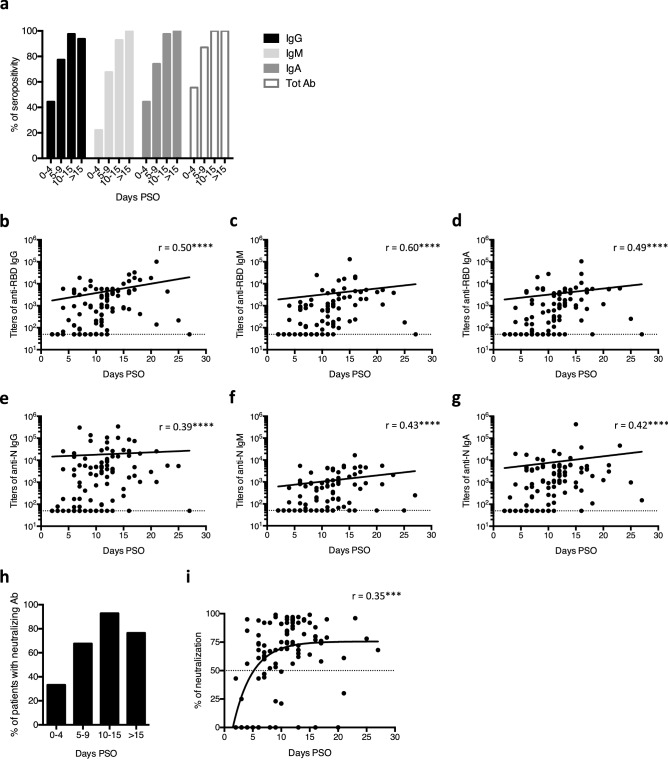
Kinetics of antibody responses against SARS-CoV-2 in hospitalized COVID-19 patients. Hospitalized COVID-19 patients include non-ICU, ICU and deceased COVID-19 patients. (**a**,**h**) Percentage of seropositivity (**a**) and percentage of patients with neutralizing antibodies (**h**) in hospitalized COVID-19 patients, according to time between symptom onset and sample collection. Seropositivity corresponds to the presence of anti-RBD and/or anti-N antibodies in serum/plasma. Serum/plasma collection was performed 0–4 days post-symptoms onset (PSO) in 9 patients, 5–9 days PSO in 31 patients, 10–15 days PSO in 42 patients and more than 15 days PSO in 16 patients. (**b**–**g**,**i**) Titers of anti-RBD IgG (**b**), IgM (**c**) and IgA (**d**), titers of anti-N IgG (**e**), IgM (**f**) and IgA (**g**) and spike-pseudotyped lentiviral neutralization (**i**) in hospitalized COVID-19 patients according to the time between symptom onset and sample collection. Each dot represents one sample. Dashed line represents the positivity threshold. The preferred model for best fit curve (solid line) was assessed by an extra sum-of-squares F test and is a semi-log line (**b**–**g**) or a one-phase association (**i**). The Spearman’s correlation coefficient (r) is shown. ***p < 0.001, ****p < 0.0001 (Spearman test).

As reflected by Fig. 1, antibody titers were found to be heterogenous within patient groups. While this may be partly attributed to individual differences in the capacity to produce anti-SARS-CoV-2 antibodies, heterogeneity of sampling times is likely also a significant factor. To analyse the kinetics of antibody production, we divided hospitalized COVID-19 (including non-ICU, ICU and deceased) patients according to the time between symptom onset and serum collection. We found that anti-SARS-CoV-2 antibodies were already detectable in 55.56% of serum collected between 0 and 4 days PSO. At day 10 PSO, all tested patients had anti-SARS-CoV-2 antibodies. The maximum percentage of seropositivity for IgG was reached between 10 and 15 days PSO, with 97.62% of hospitalized patients positive for anti-SARS-CoV-2 IgG during this period (Fig. 3a). All tested patients had anti-SARS-CoV-2 IgM and IgA from day 15 PSO. Amplitude of the antibody response increased between symptom onset and 27 days PSO, with a Spearman coefficient ranging from 0.39 for anti-N IgG to 0.6 for anti-RBD IgM (Fig. 3b–g). Of note, deceased patients present similar kinetics of anti-SARS-CoV-2 humoral response compared to the two other groups (Supplementary Fig. S1). In parallel, the maximum proportion of patients with neutralizing antibodies was found in samples taken between 10 and 15 days PSO (Fig. 3h) and neutralization capacity increased also with time (Fig. 3i).

### Persistence of anti-SARS-CoV-2 antibodies for at least 3 months

**Figure 4:**
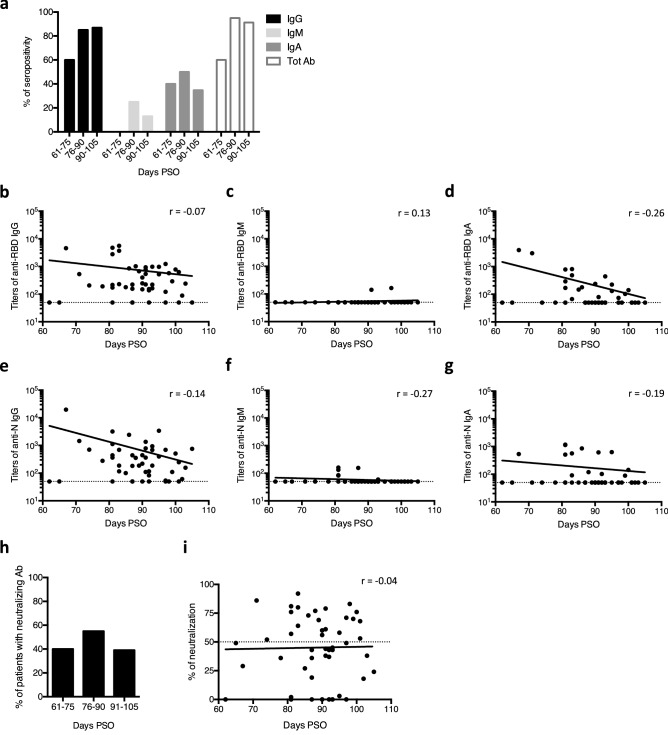
Kinetics of antibody responses against SARS-CoV-2 in recovered COVID-19 patients. Recovered COVID-19 patients were individuals infected by SARS-CoV2 more than 2 months ago who were cured. (**a**,**h**) Percentage of seropositivity (**a**) and percentage of patients with neutralizing antibodies (**h**) in recovered patients according to the time between symptom onset and sample collection. Seropositivity corresponds to the presence of anti-RBD and/or anti-N antibodies in serum/plasma. The number of patients in each group is shown in Table 1. (**b**–**g**,**i**) Titers of anti-RBD IgG (**b**), IgM (**c**) and IgA (**d**), titers of anti-N IgG (**e**), IgM (**f**) and IgA (**g**) and spike-pseudotyped lentiviral neutralization (**i**) in recovered patients according to the time between symptom onset and sample collection. Each dot represents one sample. Dashed line represents the positivity threshold. The preferred model for best fit curve (solid line) was assessed by an extra sum-of-squares F test and is a semi-log line. The Spearman’s correlation coefficient (r) is not significant for all graphs.

We studied the longevity of anti-SARS-CoV-2 antibodies and their waning in recovered patients whose serum was collected between 60 and 105 days PSO (Fig. 4). As mentioned above, titers of anti-SARS-CoV-2 IgM (RBD or N) were undetectable or very low by 60 days PSO (Fig. 4c,f). Although 86.96% of recovered patients still had anti-SARS-CoV-2 IgG 90–105 days PSO, titers of IgG tended to decrease over time (Fig. 4b,e). Anti-RBD IgA showed the same trend (Fig. 4d). Neutralizing antibodies persisted in about 40% of recovered patients and neutralization capacity remained constant over time (Fig. 4h,i).

## Discussion

In this report, we analyzed the antibody response in 187 COVID-19 patients, ranging from asymptomatic individuals to patients who died of COVID-19. We detected higher titers of virus-specific antibodies in hospitalized and deceased patients compared to asymptomatic patients. In addition, the seroneutralization capacity correlated with disease severity. Of note, higher titers of anti-SARS-CoV-2 antibodies in more severely affected patients could be due to higher viral load^31,32^ and therefore, higher antigen exposure. However, in 34 out of 100 hospitalized patients for whom SARS-CoV-2 RT-PCR Ct values were available to us, we observed no differences in these values between our different groups, nor any significant correlation between Ct values and and immunoglobulin levels (data not shown).

Our results are consistent with prior reports on SARS-CoV-2^26,33^ as well as other coronaviruses^34–36^, contrasting with reported findings of low titers of anti-RBD IgG or neutralizing antibodies in deceased patients^20^. To account for this apparent contradiction, some studies suggest that the antigen specificity rather than the quantity of antibodies may be important in infection control, showing higher anti-S/anti-N antibody ratios in humoral responses of patients with mild COVID-19 compared to patients with more severe disease^24,37^. In addition, antibody responses to the nucleocapsid were elevated in deceased individuals^37^. In our cohort, a greater proportion of asymptomatic patients had anti-RBD IgA than anti-N IgA (82.4% vs. 47.1%), whereas the reverse was observed in deceased patients (56.5% vs. 69.6%). However, the ratio between anti-RBD/anti-N IgA titers is not significantly different between these two cohorts of patients, even if it is slightly higher in asymptomatic than in deceased patients [4.79 ± 1.03 (mean ± SEM) for asymptomatic patients vs. 3.54 ± 1.03 for deceased patients]. The RBD/N ratio of IgG and IgM is also not different between asymptomatic and deceased patients, suggesting that antigen-specificity may not account for outcome in our cohort. Moreover, we show that neutralizing antibodies are positively correlated with disease severity. These were rapidly and highly produced in three quarters of deceased patients, casting some doubt on their clinical benefit, even if it has been shown that convalescent plasma therapy can control SARS-CoV-2 infection^33,38–41^. This is in contrast to the study by Lucas et al. that showed that the generation of neutralizing antibodies within 14 days of disease onset is a key factor for recovery^20^.

COVID-19 outcome has also been associated with the kinetics and isotype of antibody production rather than quantity: rapid production and higher titers of S-specific IgG were described in survivors of severe disease compared to non-survivors, indicating that rapid and potent IgG class switching is linked to survival^42^. In our study, anti-SARS-CoV-2 antibodies were rapidly detected in hospitalized individuals, including deceased patients. Between day 0 and day 4 PSO, 55.56% of hospitalized patients already had some anti-SARS-CoV-2 antibodies and 33.33% had a neutralizing action in their serum or plasma. This early seroconversion could be explained by a delayed onset of symptoms. Indeed, incubation time is 5 days on average but can be up to 14 days in some cases, allowing time to mount a humoral response. Maximal seropositivity rate was reached by 10 days PSO, since all hospitalized patients had at least one type of anti-SARS-CoV-2 antibodies at this time-point. Of note, the kinetics of anti-RBD or N IgG are similar between all our groups. In parallel, the percentage of hospitalized patients with neutralizing antibodies reached 92.9%. This demonstrates that a rapid production of anti-SARS-CoV-2 antibodies, even if neutralizing, is not sufficient to prevent severe forms of the disease.

A key clinical issue is how long anti-SARS-CoV-2 antibodies persist after infection. While some reports claim persistence of the humoral response over several months^27–29^, others show a waning of anti-SARS-CoV-2 from the first months^25,26^. In addition, cases of reinfection have been described in about 1% of patients^43–46^. We showed that more than 60 days PSO, few patients still had anti-SARS-CoV-2 IgM (< 15.1%) and IgA (< 34%), while the majority still had anti-SARS-CoV2 IgG. 88.68% of recovered COVID-19 patients had anti-RBD and/or anti-N antibodies and 45.8% had neutralizing antibodies. Of note, the neutralization capacity correlated with anti-RBD IgG titers, suggesting that these antibodies are more prone to neutralize the virus. The same observation has been made by others^24,28,47^. Although the seropositivity rate after 90 days remained higher than 85% in these patients, we showed that titers of anti-RBD IgG, anti-RBD IgA and anti-N IgG tended to decline between day 61 and day 105 PSO.

Taken together, our observations demonstrate that humoral response cannot fully explain disease course. There is controversy in the literature about the role of B cells and antibodies in virus clearance and disease course. Several studies have shown that rituximab, which leads to complete B cells deletion, is associated with a poor prognosis for COVID-19^48,49^. Conversely, another study showed that patients with agammaglobulinemia developed a mild form of COVID-19 characterised by short duration and favourable outcome without need for treatment^50^. In this case, at least, other mechanisms such as memory T cell production, must contribute to patient outcomes. Memory T cells respond better and faster upon reinfection. A recent study showed that immune memory was retained in about 95% of patients 6 months after infection^51^ suggesting that COVID-19 patients can be protected from reinfection at least this long.

Limitations of our study are the lack of paired samples and the heterogeneity in cohorts sampling. Indeed, non-ICU, ICU and deceased patients were representative of hospitalized population whereas healthy controls, asymptomatic and non-hospitalized recovered patients were enrolled among hospital staff. There is therefore a bias given the predominance of women in this setting and the fact that they are individuals of working age. A follow-up age- and gender-matched study would be of great interest to eliminate these potential cofounding factors. Another limitation is the relatively short period of analysis post infection. A better understanding of the key factors necessary for protection against reinfection as well as the duration of this protection is essential for the management of the pandemic. As more people get vaccinated, it will be necessary to determine how long vaccinated individuals may be protected, and whether boosters will be required. Longer-term studies on the immune memory induced by SARS-CoV-2 and related vaccines will be essential in addressing these practical, highly impactful issues.

## Material and methods

### Sample and data collection

All plasma/serum samples of COVID-19 patients and healthy individuals were obtained from Cliniques universitaires Saint-Luc, Brussels. The study and data collection were conducted with the approval of the hospital and faculty institutional review board (Commission d’Ethique Biomédicale Hospitalo-Facultaire) of Université catholique de Louvain, Belgium (2020/14Mai/275). All experiments were performed in accordance with relevant guidelines and regulations. Informed consent for inclusion was obtained from all participants in this study. All patient data were anonymized before study inclusion.

### Study design and participants

For this study, we included specimens from 6 cohorts of patients: healthy individuals, asymptomatic, non-ICU, ICU, deceased and recovered COVID-19 patients. Healthy individuals include 17 pre-pandemic healthy donors and 35 healthy donors recruited after December 2019. All samples from pre-pandemic healthy donors were sera. Among samples from post-pandemic healthy donors, 20 were sera and 15 were plasma. Asymptomatic patients were identified by a serological screening for anti-SARS-CoV-2 antibodies. All samples from asymptomatic patients were sera. Non-ICU patients correspond to individuals who have been hospitalized in a conventional unit for lung failure. In this cohort, 18 samples were sera, and 26 samples were plasma. Among 33 samples for ICU patients, 10 were sera and 23 were plasma. Deceased COVID-19 patients were hospitalized COVID-19 patients who died from their illness. Among 23 samples, 7 were sera and 16 were plasma. Recovered COVID-19 patients had a mild to moderate form of COVID-19 more than 2 months ago, that did not require hospitalization. All samples from recovered patients were sera. All hospitalized and recovered patients were tested positive for SARS-CoV-2 by RT-PCR on nasopharyngeal swab specimens. Clinical characteristics of each cohort are shown in Table 1.

### Enzyme-linked immunosorbent assay (ELISA)

Anti-SARS-CoV-2 antibodies were detected using in-house ELISAs. These ELISAs were performed in 96-well Maxisorp plates (ThermoFisher, 430341) coated with 50 µl per well of RBD (Genscript, Z03483), N (Genscript, Z03488) or BSA diluted at 3 µg/ml in a coating buffer (40 mM glycine, pH 9.4). After overnight incubation at 4 °C, plates were washed 3 times with a solution containing 100 mM NaCl, 20 mM TRIS HCl pH7.5 and 0.1% Tween-20. Next, 200 µl of blocking solution (PBS supplemented with 5% BSA) was added to each well for 1.5 h at 37 °C. After 3 washes, serial dilutions of serum samples (fivefold dilutions from 1/50 to 1/781250 in blocking solution) were added. Plates were incubated 2 h at 37 °C and washed 3 times. To minimize background, 200 µl of PBS supplemented with 1% NP40 was added to each well and plates were incubated 5 min at room temperature. After extensive washing, 50 µl of horseradish peroxidase (HRP)-labelled anti-human IgG (ThermoFisher, a18823), anti-human IgM-HRP (ThermoFisher, a18841), or anti-human IgA-HRP (ThermoFisher, a18787) at 500 ng/ml were added, and plates were incubated 1.5 h at 37 °C. Finally, plates were washed and loaded with 50 µl of TMB substrate solution (ThermoFisher, 34028) in each well, at room temperature. After 2 min, 50 µl of H2SO4 2 M was added to each well to stop the reaction. Optical density (OD) at 450 nm was measured using a microplate reader spectrophotometer (VERSAmax; Molecular Devices, San Jose, California). The OD value from the BSA-coated wells was subtracted from the OD of the antigen (RBD or N) coated wells. Titers of anti-N IgG correspond to 50% of the maximal OD while titers of anti-RBD IgG, anti-N IgM, anti-RBD IgM and anti-N IgA correspond to 25% of the maximal OD. Titers of anti-RBD IgA correspond to 10% of the maximal OD. The cut-offs for each ELISA were established to achieve maximal sensitivity and specificity of anti-SARS-CoV2 antibody detection.

### Generation of lentiviruses

Lentiviral vectors were derived from pTM897^52^, a derivative of pCCLsin.PPT.hPGK.GFP.pre.^53^ in which a multiple cloning site was inserted downstream of the phosphoglycerate kinase (PGK) promoter. pTM914 and pMK02 were constructed by cloning, in pTM897, the firefly luciferase coding region of pGL3 (Promega) and the Renilla luciferase coding region of pGL4.78 (Promega), respectively. pTM900 is a derivative of pTM897 carrying an IRES-Hygromycin resistance gene cassette cloned from pQCXIH (Clontech). The ACE2 coding sequence was PCR-amplified from pACE2-eGFP (kind gift of D. Alsteens, LIBST, UCLouvain) and cloned into pTM900 to yield pTM1148.

A SARS-CoV-2 spike carrying a C-terminal deletion of 19 residues (SARS-CoV-2 SpikeΔC19) was used for pseudotyping as this deletion was shown to increase pseudotyping efficiency^54^. Therefore, a synthetic gene coding for the spike protein of SARS-CoV-2, strain Wuhan-Hu-1 (accession YP_009724390.1) kindly provided by A. Nedelec and S. Constantinescu (Ludwig Institute, UClouvain) was mutated to introduce the ΔC19 mutation and cloned in pcDNA3 (ThermoFisher) to generate pTM1156.

Lentivirus particles were generated by transient transfection of 293 T cells grown in 75-cm^2^ Petri dishes by the calcium phosphate method, using 7.5 μg of lentiviral vector, 3.375 μg of pMDLg/pRRE (Gag-Pol), 1.875 μg of pRSV-Rev (Rev), and 2.25 μg of either pMD2-VSV-G (VSV-glycoprotein)^53^ or pTM1156 (SARS-CoV-2 SpikeΔC19). Cell supernatants harvested at 48 or 72 h post transfection were passed through a 0.45-um filter. SARS-CoV-2 SpikeΔC19-pseudotyped lentiviral particles were purified and concentrated by centrifugation at 10,000*g* for 4 h at 4 °C through a 10% sucrose cushion (sucrose 10% w/v in 50 mM Tris–HCl pH7.4; 100 mM NaCl; 0.5 mM EDTA) in oak-ridge tubes containing a 4/1 ratio of lentivirus containing supernatant (typically 16 ml) and of sucrose solution (typically 4 ml).

The following lentivirus stocks were used in this study: the Firely luciferase pseudotyped with SpikeΔC19, the Renilla luciferase pseudotyped with vesicular stomatitis virus (VSV) glycoprotein and the ACE2-IRES-Hygro^r^ pseudotyped with VSV glycoprotein.

Firefly luciferase activity measured 72 h after transduction of cells with SpikeΔC19-pseudotyped was > 20-fold higher in 293 T-ACE2 than in 293 T cells, confirming ACE2-dependent transduction. It is noteworthy, however, that transduction after pseudotyping with SARS-CoV-2 SpikeΔC19 was 59,000 (± 10,000)-fold less efficient than pseudotyping with VSV glycoprotein.

### Seroneutralization assay

293 T cells^55^ were grown in DMEM medium (Lonza) supplemented with 10% fetal calf serum and 100 u/ml of penicillin and streptomycin (DMEMc). 293 T-ACE2 cells were obtained by transduction of 293 T cells with TM1148. Cells were selected with 200 μg/ml of hygromycin (Roche) and tested by western blot for ACE2 expression. 293 T-ACE2 cells were then seeded in 96-well plates at a density of 2000 cells/well in 100 µl of DMEMc medium. 24 h later, sera to be tested or control antibodies were incubated at indicated dilutions for 1 h at 37 °C in 60 μl of OPTI-MEM medium (ThermoFisher) containing SpikeΔC19-pseudotyped (4–6 μl) and VSVg-pseudotyped (0.2 μl) reported lentiviruses. A monoclonal neutralizing antibody (Sino Biological, 40591-MM43) was used as a positive neutralization control. Lentivirus concentrations in the mix were adjusted from batch to batch to yield detectable luciferase activity. The mixes containing the lentiviral vectors incubated with the sera were then transferred to the wells seeded with 293 T-ACE2 cells. After 72 h, cells were lysed and transduction efficiency was measured using a dual luciferase assay (Promega).

### Statistics

Results are presented as the mean ± SD. Statistical significance between groups was assessed by using one-way Kruskal–Wallis test with Dunn’s multiple comparisons test. Correlations were assessed using linear regression and Spearman’s test. For kinetic analyses, the best fitting curve was defined by an extra sum-of-squares F test, selecting the simpler model unless *p* < 0.05. Semi-log line, one-phase association and two-phase association were evaluated in all cases. Statistics were performed with the Prism sofware (GraphPad sofware).

## Supplementary Information

Supplementary Information.

## Data Availability

The datasets generated and/or analysed during the current study are available from the corresponding author on reasonable request.
