# Supplementary material for: Development of SARS-CoV2 humoral response including neutralizing antibodies is not sufficient to protect patients against fatal infection
Source: Sci Rep. 2022 Feb 8;12:2077. doi: 10.1038/s41598-022-06038-5 (PMC8827092; doi:10.1038/s41598-022-06038-5)
Supplement: Supplementary file 1 — Supplementary Information. [file 41598_2022_6038_MOESM1_ESM.docx]

**Development of SARS-CoV2 humoral response including neutralizing antibodies is not sufficient to protect patients against fatal infection**

Mathilde Choteau^1^, Anaïs Scohy^2,3^, Stéphane Messe^4^, Mathieu Luyckx^5,6^, Mélanie Dechamps^7,8^, Virginie Montiel^9^, Jean Cyr Yombi^3,10^, Damien Gruson^2,3^, Nisha Limaye^11^, Thomas Michiels^4^, and Laure Dumoutier^1*^

**Affiliations**

^1^ Experimental Medicine Unit, de Duve Institute, Université catholique de Louvain, Brussels, Belgium

^2^ Department of Laboratory Medicine, Cliniques Universitaires Saint-Luc, Brussels, Belgium

^3^ Institut de Recherche Expérimentale et Clinique (IREC), Université catholique de Louvain, Brussels, Belgium

^4^ Virology Unit, de Duve Institute, Université catholique de Louvain, Brussels, Belgium

^5^ Department of Gynecology-Andrology, Cliniques Universitaires Saint-Luc, Université Catholique de Louvain, Brussels, Belgium

^6^ Tumor Infiltrating Lymphocytes Unit, de Duve Institute, Université catholique de Louvain, Brussels, Belgium

^7^ Pôle de Recherche Cardiovasculaire (CARD), Institut de Recherche Expérimentale et Clinique (IREC), Université catholique de Louvain (UCLouvain), Brussels, Belgium

^8^ Cardiovascular Intensive Care, Cliniques Universitaires Saint-Luc, Brussels, Belgium

^9^ Intensive Care, Cliniques Universitaires Saint-Luc, Brussels, Belgium

^10^ Department of Internal Medicine and Infectious Diseases, Cliniques universitaires Saint-Luc, Brussels, Belgium

^11^ Genetics of Autoimmune Diseases and Cancer Unit, de Duve Institute, Université catholique de Louvain, Brussels, Belgium

* contact person :

Laure Dumoutier: laure.dumoutier@uclouvain.be

De Duve Institute - MEXP, cytokine group

74 avenue Hippocrate

B-1200 Brussels

+32 2 7647465

**Supplementary Figure S1.** Kinetics of anti-SARS-CoV-2 antibody production in non-ICU, ICU and deceased COVID-19 patients.

Non-ICU

ICU

Deceased

**a**

**b**

**c**

**d**

**e**

**f**

RBD

N

*

*

*

*

**

Titers of anti-RBD **(a, c, e)** and anti-N antibodies **(b, d, f)** in non-ICU **(a, b)**, ICU **(c, d)** and deceased **(e, f)** COVID-19 patients according to the time between symptoms onset and sample collection. The number of samples in each group is shown in Table 1. Dashed line represents the positivity threshold. **p* < 0.05, ***p* < 0.01 (Kruskal Wallis test and Dunn’s multiple comparisons test).
